# Choreoathetosis – an unusual adverse effect of dihydroartemisinin-piperaquine: a case report

**DOI:** 10.1186/s13256-017-1525-7

**Published:** 2017-12-28

**Authors:** Benjamin Momo Kadia, Christian Morfaw, Armelle Corrine Gounoue Simo

**Affiliations:** 1 Foumbot District Hospital, Foumbot, Cameroon; 2 West Region Technical Group for the fight against HIV/AIDS, ᅟBafoussam, Cameroon; 3 Faculty of Health Sciences, University of Buea, Buea, Cameroon; 4 Clinical Research Education, Networking and Consultancy, Douala, Cameroon

**Keywords:** Dihydroartemisinin-piperaquine, Choreoathetosis, Case report

## Abstract

**Background:**

Dihydroartemisinin-piperaquine is a combination of dihydroartemisinin and piperaquine which is highly effective in the treatment of uncomplicated falciparum malaria. Its adverse effects are generally tolerable and temporary. Choreoathetosis, an involuntary movement disorder characterized by continuous irregular twisting of the body, is not a documented adverse effect of this medication.

**Case presentation:**

A 41-year-old Cameroonian man of black African ethnicity was brought to our primary care hospital because over the previous 6 hours he had been experiencing involuntary twisting movements of his body and he no longer had control of his limbs. Earlier that day, he had been prescribed an appropriate dose of dihydroartemisinin-piperaquine in our hospital. The abnormal movements started approximately 3 hours after ingesting the first dose of the drug. The review of systems and his past history were unremarkable. On clinical examination, he was conscious and oriented but was unsteady and displayed continuous generalized irregular twisting movements combined with abrupt low amplitude flinging of his limbs. Dihydroartemisinin-piperaquine-induced generalized choreoathetosis was diagnosed. He was sedated with diazepam and dihydroartemisinin-piperaquine was discontinued. The antimalarial drug was substituted with artemether-lumefantrine combination. The clinical progress was good and he was discharged home after 72 hours. No further abnormalities were noted during 7 months of follow-up.

**Conclusion:**

Although dihydroartemisinin-piperaquine is increasingly popular as a well-tolerated/efficacious antimalarial drug, clinicians must note the rare possibility of choreoathetosis as an adverse effect of this medication and educate patients accordingly.

## Background

Choreoathetosis is an involuntary movement disorder characterized by continuous, non-rhythmic, irregular throwing and writhing of the body which can be focal or generalized. It has diverse etiologies including genetic and hereditary disorders, cerebrovascular disease, poststreptococcal infection, connective tissue diseases, thyroid disorders, hyperglycemia, and post-traumatic brain injury [1]. With regards to pharmacologic agents, neuropsychiatric drugs have been most implicated in the cause of choreoathetosis. Very few antimicrobials have been associated with the onset of this movement disorder.

Dihydroartemisinin-piperaquine (DHA-PPQ) is a fixed-dose combination of dihydroartemisinin (DHA) 40 mg and piperaquine (PPQ) 320 mg which is very effective in the treatment of uncomplicated falciparum malaria [2–4]. The reported adverse effects of this drug are generally tolerable and temporary and they include dizziness, headache, cough, nausea, vomiting, anorexia, asthenia, abdominal pain, diarrhea, fever, as well as changes in biochemical and blood indices [5]. This medication can also lead to prolonged QT interval with irregularities in heart rhythm that may be fatal [6]. Choreoathetosis is not a documented side effect of this medication. We, however, report a case of possible DHA-PPQ-associated acute generalized choreoathetosis.

## Case presentation

A 41-year-old Cameroonian man of black African ethnicity was brought to our primary care hospital because over the previous 6 hours he had been experiencing persistent involuntary twisting movements of his body and he no longer had control of his limbs. Earlier that day, he had presented to our hospital with low-grade intermittent fever of nocturnal predominance associated with headache, anorexia, and mild general weakness for 3 days. On that occasion, he was mildly ill-looking and calm. He was alert and oriented in person, place, and time. His vital signs were: temperature, 38.7 °C; blood pressure, 122/83 mmHg; pulse, 91 beats/minute (full and regular); and respiratory rate, 17 breaths/minute. His conjunctivae were pink. His neck was supple. There were no palpable enlarged lymph nodes. His cranial nerve functions as well as deep tendon and cutaneous reflexes were symmetrical and normal. His muscle bulk was good and his muscle tone was normal. Muscle strength was full force. He could execute coordinated movements and he had a normal gait. There was normal sensation to fine touch, crude touch, and vibrations. There was no focal neurological sign and both plantar reflexes were up-going. Chest wall expansions were symmetrical and the cardiac apex beat was located at the left fifth intercostal space in the midclavicular line. Percussion note on his chest wall was resonant. Breath sounds were vesicular and there were no heart murmurs. His abdomen was symmetrical and moved with respiration. There was no abdominal tenderness and no palpable enlarged abdominal organs. The percussion note on his abdomen was tympanic. Bowel sounds were active and normal. The rest of systemic examination was unrevealing as well. Based on those findings, he was presumptively diagnosed with simple malaria. Moderate *Plasmodium falciparum* parasitemia was found on microscopy (blood film analysis). He was prescribed DHA-PPQ at a dose of 2.1/16.8 mg/kg (total of three tablets of DHA 40 mg-PPQ 320 mg) in a single daily dose and 2 g of paracetamol tablets in two divided doses per day. These dosages were estimated for his 71 kg body weight and both drugs were prescribed for 3 consecutive days. Approximately 3 hours after ingesting the first dose of the drug, there was progressive onset of the bizarre movements which were rather amusing to his brother who first thought our patient was joking. However, the movements which were initially intermittent progressively became continuous.

Our patient was not on any other medication prior to the diagnosis of malaria and he had never experienced abnormal movements before. His past history was negative for known chronic illnesses like hypertension, diabetes, neuropsychiatric disorders, chronic kidney disease, or cancer. He had no history of head trauma or neurological disorders like encephalitis or cerebrovascular accident. He had no known history of rheumatic fever. He had no known records of adverse reactions to specific drugs or food and as far as he could remember, he had not ingested DHA-PPQ before. He did not consume tobacco or recreational drugs including traditional medicines. He seldom consumed beer and did not smoke tobacco. He had been a subsistence farmer for 20 years. He lived in the city center and consumed pipe-borne water. He had no known history of exposure to environmental hazards such as pesticides, industrial wastes, or other toxic chemicals at home or at work. He was married and had three children. His wife and children were well. He was fifth in a family with four siblings; his siblings and parents were well. His family history was positive for first-degree relatives with hypertension but negative for movement disorders and other chronic illnesses. There were no known genetic disorders in his family.

On his second visit, a systemic review was unremarkable. His Glasgow Coma Score was 15/15 and he was oriented in person, place, and time. His blood pressure was 133/87 mmHg, temperature 37.4 °C, and pulse 83 beats/minute. He was very unsteady (making his respiratory rate difficult to appreciate) and displayed continuous irregular twisting movements of his face, neck, extremities, and trunk combined with abrupt forceful low amplitude flinging of his limbs. These movements were precipitated and exacerbated by any voluntary attempts to move such that he could not execute purposeful movements requested by the examiner. Examination of his cranial nerves and grading of muscle strength were difficult because he was unsteady. Muscle tone as well as deep tendon and cutaneous reflexes were normal. The rest of the physical examination was normal.

In view of the historical and clinical data, DHA-PPQ-associated acute generalized choreoathetosis was suspected and dystonia was retained as differential diagnosis. Further laboratory investigations such as complete blood count, blood electrolytes, and tests of renal function were without particularities (Table 1). He was sedated with 10 mg of intramuscular diazepam and DHA-PPQ was discontinued. The antimalarial drug was replaced with artemether-lumefantrine combination (20 mg to 120 mg strength – single initial dose of four tablets, followed by four tablets after 8 hours and then four tablets 12 hourly to reach a total of 24 tablets) which was better tolerated. His clinical progress was good and he was discharged home after 72 hours. No further abnormal movements were noted during 3 weeks of regular follow-up. Monthly reviews over the following 7 months were unrevealing.

**Table 1:** Laboratory test results of the patient

Laboratory tests	Results	Interpretation/Conclusion	After 3 weeks
Microscopy		Moderate *Plasmodium falciparum* parasitemia	No observable parasites
Thick blood smear	7.3 × 10^4^ trophozoites/mcL		
Thin blood smear	*Plasmodium falciparum*		
Full blood count		Normal	
White cell indices			
Total white cell count	9000/mcL		
Neutrophils	5210/mcL		
Lymphocytes	3700/mcL		
Monocytes	83/mcL		
Red cell indices			
Red cell count	4.7 million/mcL		
Hematocrit	41%		
Mean cell volume	83fL		
Platelets	356,000/mcL		
Blood electrolytes		Normal	
Sodium	140 mEq/L		
Potassium	4.1 mEq/L		
Calcium	9.3 mg/dL		
Magnesium	2 mg/dL		
Chloride	101 mEq/L		
Serum creatinine	0.9 mg/dL	Normal	
Liver enzymes		Normal	
Alanine aminotransferase	23 IU/L		
Aspartate aminotransferase	21 IU/L		
Serological tests		Negative	
For HIV	Negative		
For hepatitis B and C	Negative		
VDRL	Non-reactive		
Fasting blood sugar	116 mg/dL	Normal	
Urine analysis	Without particularity	Without particularity	
C-reactive protein	4 mg/dL	Within normal limits	

*fL* femtoliter, *IU/L* International Units per liter, *mcL* microliter, *mEq/L*, milliequivalent per liter, *mg/dL* milligrams per deciliter, *VDRL* Venereal Disease Research Laboratory

## Discussion

To the best of our knowledge, this is the first reported case of DHA-PPQ-associated acute generalized choreoathetosis. Dystonia was considered a differential diagnosis but the swinging of limbs, and the lack of significant abnormalities in posturing/muscle tone made this differential less likely in the case presented. Tardive dyskinesia, rheumatic fever (Sydenham’s chorea), and unusual causes of choreoathetosis like endocrine disorders and paraneoplastic syndrome were less plausible etiological differentials given our patient’s historical and clinical data, in particular, the temporal relationship between ingestion of DHA-PPQ and onset of choreoathetosis. Furthermore, non-resurgence of the movement disorder after the withdrawal of DHA-PPQ and switching to artemether-lumefantrine lends more credence to a diagnosis of possible DHA-PPQ-induced choreoathetosis. Despite the positive outcome, the case report was flawed by our inability to perform a controlled drug challenge test or measure serum concentrations of DHA-PPQ in order to ascertain that the observed adverse effect was truly unrelated to drug dosage.

Falciparum malaria remains a serious public health concern which is increasingly compounded by the problem of resistance of *Plasmodium falciparum* to traditional antimalarial drugs like chloroquine and sulfadoxine-pyrimethamine [7]. In order to counter the spread of resistance, the World Health Organization (WHO) recommends the use of artemisinin-based combination therapies (ACTs) as first-line treatment of uncomplicated falciparum malaria [8, 9]. ACTs are preparations of short-acting artemisinin derivatives (like artesunate, artemether, and DHA) partnered with long-acting drugs. In principle, if a parasite mutation that leads to drug resistance arises during treatment, the parasite should be killed by the long-acting drug, thus limiting the development of resistance [10].

In line with the recent WHO recommendations, various ACTs have been designed and are currently in use on a global scale. One of these is DHA-PPQ. DHA is the active metabolite of the artemisinin derivatives which produces faster relief of clinical symptoms and faster clearance of the parasites from blood when compared with other antimalarial drugs [9]. PPQ, which is a 4-aminoquinoline like chloroquine, has a very long elimination half-life and could provide a long period of post-treatment prophylaxis [11]. Accumulating evidence suggests that the efficacy of DHA-PPQ is comparable to that of commonly used first-line ACTs like artemether-lumefantrine [7, 12].

Although concerns have been raised regarding animal neurotoxicity [13], artemisinin derivatives such as DHA have been used safely in a large number of patients with uncomplicated or severe malaria [14]. PPQ has been used less widely, but studies suggest that it is as potent as chloroquine but less toxic [15, 16]. However, in a recent randomized controlled trial involving 150 patients, Gargano *et al*. noted an unusual case of DHA-PPQ-induced grand mal convulsions 3 hours after the first appropriate dose of the drug had been administered [17] and this suggests that in rare cases, DHA-PPQ may possibly trigger adverse neurological events. Taking these into account, the unusual incidence of choreoathetosis which we noted in our patient who had ingested an appropriate dose of DHA-PPQ, may suggest an idiosyncratic origin of this movement disorder and possibly other rare neurological complications.

An idiosyncratic drug reaction (IDR) refers to an adverse reaction that does not occur in most patients treated with a drug, does not involve the known pharmacologic properties of that drug, and does not show any apparent dose-response relationship [18]. While noting that IDR characteristically has a delay in onset, rapid onset IDR (as may have been the scenario in our case) is possible [19]. Choreoathetosis in our patient may have been consequent to off-target pharmacology which has been proposed as an idiosyncratic mechanism in many unusual central nervous system adverse drug reactions [18]. In this regard, previous rare reports on drug-induced movement disorders highlight possible roles of imbalanced gamma-aminobutyric acid (GABA) receptor activity, cytokine release, and excessive glutamate which may contribute to altered excitatory and inhibitory cortical and subcortical motor control pathways [20]. Even though these suggestions and the chemical properties of a drug may explain the onset of movement disorders, the underlying patient factors that contribute in individual susceptibility to such rare drug-induced movement disorders are generally poorly elucidated.

## Conclusions

We report a rare case of possible DHA-PPQ-induced choreoathetosis in a black African man who had ingested an appropriate daily dose of the drug. Despite the popular use of DHA-PPQ in the treatment of uncomplicated falciparum malaria and the growing consensus on its efficacy/tolerability, clinicians must note the rare possibility of this central nervous system adverse effect and educate patients accordingly.

## Abbreviations

ACTs: Artemisinin-based combination therapies
DHA: Dihydroartemisinin
DHA-PPQ: Dihydroartemisinin-piperaquine
IDR: Idiosyncratic drug reaction
PPQ: Piperaquine phosphate
WHO: World Health Organization
